# Social Isolation Affects the Mimicry Response in the Use of Smartphones: An Ethological Experiment during the COVID-19 Pandemic

**DOI:** 10.1007/s12110-023-09443-5

**Published:** 2023-02-21

**Authors:** Veronica Maglieri, Anna Zanoli, Dimitri Giunchi, Elisabetta Palagi

**Affiliations:** 1 Unit of Ethology, Department of Biology, University of Pisa, Via Volta 6, Pisa, 56126 Italy; 2 Department of Life Sciences and Systems Biology, University of Turin, Turin, Italy

**Keywords:** Social isolation, Chameleon effect, Gaze-following, Joint actions, Social bonds

## Abstract

Humans are social animals that rely on different ways to interact with each other. The COVID-19 pandemic strongly changed our communication strategies. Because of the importance of direct contact for our species, we predict that immediately after the forced social isolation, people were more prone to engage in direct rather than in virtual interactions, thus showing a lower mimicry response in the use of smartphones. In a non-longitudinal study, we collected behavioral data under naturalistic contexts and directly compared the data of the mimicry response gathered immediately following the Italian lockdown (May–September 2020) with those gathered one year later (May–October 2021). Contrary to our expectations, the mimicry response in the use of smartphones was higher immediately after the lockdown than a year later. Probably the large use of these devices during the lockdown translated into a greater sensitivity to be affected by others’ smartphone manipulation. Indeed, social isolation modified, at least in the short term, the ways we interact with others by making us more prone to engage in “virtual” social interactions. The bright side of the coin unveiled by our findings is that the effect seems to diminish over time. The large behavioral dataset analyzed here (1,608 events; 248 people) also revealed that the mimicry response in the use of smartphones was higher between familiar subjects than between strangers. In this view, mimicry in manipulating smartphones can be considered an example of joint action that fosters behavioral synchrony between individuals that, in the long-term, can translate into the formation of social bonding.

Primates are social animals that live in complex societies and form social bonds with conspecifics by engaging in social grooming, which triggers the endorphin system (Keverne et al., 1989; Nummenmaa et al., 2016; Sutcliffe et al., 2012). Since the time available to groom conspecifics is not unlimited, this sets a bound for individual grooming networks which in some species can reach 50 individuals (Dunbar, 2003). According to the grooming-at-distance hypothesis (Dunbar, 2003), our species is able to manage larger groups (reaching a maximum of 150 individuals) because we evolved different strategies to “groom” many individuals simultaneously (Dunbar, 2020). This has been possible by switching from “touch-based” to “virtual” grooming, which does not need any physical contact but is still capable of triggering the endorphin system (Nummenmaa et al., 2016). Humans engage in touch-based grooming with individuals who are closest to them (such as family members and partners) and reach a wider circle of acquaintances and friends thanks to virtual grooming involving less personalized interactions, such as laughing, dancing, singing, and religious rituals (Dunbar, 1998, 2022; Pearce et al., 2015) proposed that language has evolved to accommodate larger social group sizes, allowing an individual to interact with several people at once to exchange information. This can be also reflected in virtual social interactions such as texting or interacting with others through social media.

Another evolutionary mechanism that plays a role in forming and maintaining social bonds at a distance is mimicry (Chartrand & Lakin, 2013). People mimic individuals who are close to them, such as kin and friends, more than they do acquaintances and strangers (Bourgeois & Hess, 2008; Hess et al., 2022; Likowski et al., 2008; McIntosh, 2006; Palagi et al., 2020; Tickle-Degnen, 2006; Yabar et al., 2006). Facial expressions, gestures, postures, and vocal accents can evoke a mimicry response in others (Chartrand & Lakin, 2013; Genschow et al., 2017; Giles et al., 1991; Herrmann et al., 2011; Hess & Fischer, 2013, 2022; La France, 1982; Palagi et al., 2020; Tiedens & Fragale, 2003). Mimicry can also involve manipulation of objects such as pens and cigarettes (Harakeh & Vollebergh, 2012; Harakeh et al., 2007; Stel & Vonk, 2010; van Baaren et al., 2006). A recent study also demonstrates that the use of smartphones can trigger a mimicry response in the observer, and this phenomenon may be one of the reasons for the wide diffusion of these devices (Maglieri et al., 2021). After collecting ethological data on unaware people, the authors found that subjects mimicked others looking at their phones in about 50% of cases.

In 2021, 79.23% of the world’s population owned a smartphone (6,259 billion people), with forecasts suggesting that the number will likely increase to 6,567 billion by the end of 2022 (Statista, 2022). Parasuraman et al. (2017) interviewed 409 subjects to understand how people use their mobile phones, and most of the responders (87.8%) affirmed that they use smartphones for communication. In this view, smartphones could be viewed not as interfering with social activities but as increasing the number of virtual social interactions, thus reaching more individuals at the same time by texting them, liking their photos on Instagram, or reacting to their posts on Facebook. This hypothesis agrees with the data showing that during the pandemic lockdown, when direct social interactions were precluded, the number of internet users and, consequently, the number of smartphone users strikingly increased (International Telecommunications Union, 2021; Ratan et al., 2021). However, recent studies showed that even if humans benefit from virtual social interactions, face-to-face remained the only mode of contact associated with higher levels of well-being and with the capacity to properly recognize others’ emotional expressions (Kastendieck et al., 2022; Marini et al., 2021; Newson et al., 2021). Moreover, using social media which provides low levels of social presence correlates negatively with a sense of social connectedness, thus leading, in the long-term, to a sense of loneliness (Nguyen et al., 2021).

The present study aims to evaluate the effect of the March 9–May 18, 2020, Italian lockdown (DPCM issued by the Italian government on March 8, 2020; https://www.gazzettaufficiale.it/eli/gu/2020/03/08/59/sg/pdf) on the mimicry response in the use of smartphones. Specifically, we predict that immediately after the forced social isolation period, Italian people were more prone to engage in live social interactions rather than in virtual ones, thus showing a lower mimicry response in the use of smartphones. We followed the same naturalistic procedure of behavioral data collection applied by Maglieri et al. (2021). After the administration of experimental (*manipulating and looking at the screen*) and control stimuli (*manipulating without looking at the screen*), we assessed the presence of spontaneous mimicry response in the observers and directly compared the data gathered during the months immediately following the lockdown (May–September 2020) with those gathered one year later (May–October 2021).

## Materials and Methods

### Ethics Statement

The Committee on Bioethics of the University of Pisa approved the present study (Review No. 5/2020; AOO “CLE” - Prot.: 0036356/2020 of 10/04/2020). The study was purely observational, and data were entered in an anonymous form (an alphanumerical code has been uniquely assigned to each subject). People were observed in their natural social setting, not involving any interference in their spontaneous daily activities. The authors declare no conflict of interest.

### Participants and Data Collection

The dataset used for the analyses included the data collected in 2020 from Maglieri et al. (2021) and a new set of data collected in Italy across six months (May–October 2021). The 2020 data collection was carried out immediately after the Italian lockdown, a complete confinement which produced a state of isolation instituted for health security reasons (March 9–May 18, 2020). The 2021 data collection occurred exactly one year later and followed the COVID-19 regulations issued by the Italian government in 2021.

The data collection was distributed across morning (07:00 am–01:00 pm), afternoon (01:00–07:00 pm), and night (07:00 pm–03:00 am). Subjects were observed in their natural social settings during their daily activities (e.g., at work, in restaurants, waiting rooms, swab lines (COVID testing stations), social meals, and family environments) by two experimenters (co-author VM and a field assistant). To exclude the possibility of an audience effect (Tennie et al. 2010), all the subjects were unaware of being observed (blind data collection). Some of the observed subjects knew each other or the experimenters; others did not. We observed 187 subjects (93 women and 94 men). The number of people present during each observation session was recorded.

**Fig. 1:**
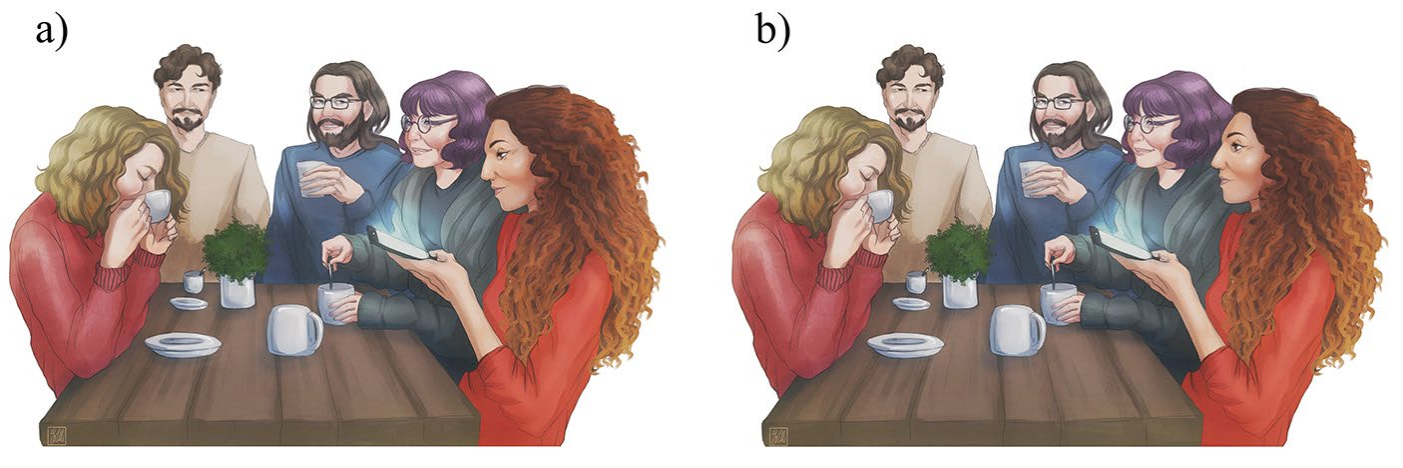
(a) Illustration of the Experimental Condition: the trigger is the person who takes and handles the smartphone for at least 5 s while looking at the screen (woman on the right). (b) Illustration of the Control Condition: the trigger takes and handles the smartphone for at least 5 s without looking at the screen (woman on the right). The two conditions differ only in the gaze of the trigger, which is directed to the device in the Experimental Condition (a) and not in the Control Condition (b). In both the conditions the device is unlocked, and the screen is turned on and visible

We included in the analysis only the sequences of actions that fulfilled the following criteria for both the Experimental (EC) and the Control (CC) conditions, as defined in Maglieri et al. (2021). The EC and CC were identical except for the presence of the action “*looking at the screen*.” In the EC, the trigger picked up, kept in their hands, handled their smartphone (e.g., fiddling and swiping), and looked at the screen for at least five seconds (Fig. 1a). In the CC, the trigger picked up, kept in their hands, and handled their smartphone (e.g., fiddling and swiping) *without* looking at the screen for at least five seconds (Fig. 1b). In both EC and CC, the trigger had to unlock the device, and the screen’s light had to be always visible. The ECs and CCs were randomly administered, and two consecutive observation bouts were separated by at least 10 min. The experimenters (VM, female; a field assistant, male) acted as triggers. We opportunistically collected data when other people (males and females) spontaneously handled/looked at their own smartphones for at least five seconds, thus acting as unaware triggers.

The observer, who had their smartphone within reach, had to visually perceive the trigger’s action, thus having the opportunity to mirror the action of the trigger in both the EC and the CC. During both conditions, the gaze of the observers had to be always visible to the experimenters. Immediately after the trigger picked up their device (t_0_), all the observers were followed for 30 s by the experimenters, who assessed the presence/absence of a mimicry response with the aid of a wristwatch (Casio F-91 W-1YER-P). The time window was set according to the results obtained by Maglieri et al. (2021), showing that the mimicry responses concentrated in the first 30-sec block of observations. In the case of mimicry response, we also recorded which kind of activity the observer did on his/her smartphone (e.g., check social media, take photos, surf the internet). It was possible to assess the activity thanks to the easily recognizable social media format. In those cases in which the experimenters could not see the observers’ screen, they directly asked for information (e.g., “what are you doing?” “what are you looking at?”).

Before starting the data collection, the two experimenters (VM and the field assistant) tested their reliability rates during 20 observational sessions by concurrently gathering data on the same observers. We calculated the Cohen’s kappa values (κ) for (1) the number and the identity of the observers and (2) the presence/absence of the mimicry event. For all these conditions, the κ values were always higher than 0.92 (Kaufmann and Rosenthal, 2009).

After 30 s, the experimenters moved away from the observers and recorded their observation on their smartphones or on paper, thus masking the action from the observed people. The observers’ identities were coded with alphanumerical strings.

We excluded from our data the cases in which people, while using their smartphones, actively solicited the observers’ attention either nonverbally or verbally.

### Operational Definitions

In both conditions (EC and CC), we recorded the presence/absence (binomial data) of the mimicry response of the observer during a 30-second time window after perceiving the trigger’s action. Response latency was defined as the time interval between the trigger first touching their smartphone and the observer’s first touching their own smartphone (see Maglieri et al. 2021).

We use the time of the day (morning; afternoon; night), sex (males and females), and the age of the trigger and observer (18–25 years; 26–40 years; 41–60 years) as categorical variables.

The relationship between the trigger and the observer was classified according to four categories: people who had never met before (strangers), people who exclusively shared an indirect relationship based on a third external factor—work duty, colleagues, friends in common, friends-of-friends (acquaintances); unrelated subjects sharing a direct friendship relationship (friends); and family members and cohabitants (partners/kin). The experimenters, in most cases, knew the relationship shared by the observed people. Personal information (e.g., relationship and age) was gathered via a friendly conversation when the trigger was different from and unknown to the experimenters. When such information was not available, we excluded the observation from the dataset.

The social contexts were also categorized according to the absence or presence of food (from when the subjects sat down at the table to when they left the table). The context “presence of food” consisted of breakfasts, lunches, dinners, and happy hours. The context “absence of food” includes the other social contexts in which food was not present (e.g., working, relaxing, playing board games or card games, waiting in a sitting room or in swab lines). In all contexts of observation, subjects always had the opportunity to handle their devices. EC and CC were randomly distributed across all possible contexts.

### Data Analyses

From May to October 2021, we collected 887 observations (*N*_EC_ = 455; *N*_CC_ = 432) of 187 subjects (93 women, 94 men). To test our hypothesis, we combined our new dataset with the dataset used by Maglieri et al. (2021) collected during the period after the Italian lockdown (May–September 2020). The new dataset contained 1,608 observations of 248 subjects (*N*_males_ = 126; *N*_females_ = 122). About 60% of the subjects were observed during both data collections. Only those subjects that were observed in both the experimental and control conditions were included in the analysis. Statistical analyses were conducted using R programming language (R Core Team, 2021).

To document the presence of a mimicry phenomenon in the use of smartphones within 30 s of the stimulus perception and to understand which factors could influence it, we built a generalized linear mixed model (GLMM) with a binomial error distribution (R-package *glmmTMB* 1.2.5042; Brooks et al., 2017). The response variable was the presence of mimicry (absence/presence), and the fixed factors were the condition (EC; CC), the sex (male; female) and age (18–25 years; 26–40 years; 41–60 years) of the observer and of the trigger, the context (presence/absence of food), the time of day (morning, afternoon, and night), the level of familiarity shared by the trigger and the receiver (strangers, acquaintances, friends, partners/family), and the pandemic period (2020, 2021). The interaction between the identities of the trigger and the observer was included as a random factor. To exclude the occurrence of collinearity among predictors, we examined the variance inflation factors (VIF; Fox, 2016) by means of the R-package *performance* 0.4.4 (Lüdecke et al., 2020). No collinearity has been found between the fixed factors (range VIF_min_=1.04; VIF_max_=1.36).

We tested the overall significance of the full model, comparing it with the null model (which included the random effects; Forstmeier and Schielzeth, 2011) by means of the likelihood ratio test (LRT; Dobson, 2002). The LRT was also used to test the significance of the fixed factors using the *Anova* function in the R-package *car* 3.0–10 (Fox & Weisberg, 2019). Model fit and overdispersion were checked using the R-package *DHARMa* 0.3.3.0 (Hartig, 2020). Marginal R², which represents the variance explained by fixed factors only, and conditional R², which represents the variance explained by the entire model, including fixed and random effects (Nakagawa et al., 2017), were calculated using the R-package *MuMIn* 1.43.17 (Bartoń, 2020).

To quantify the modality in the use of the phone by the observers in both the EC and CC we applied the chi-squared test.

## Results

### The Mimicry Phenomenon

**Fig. 2:**
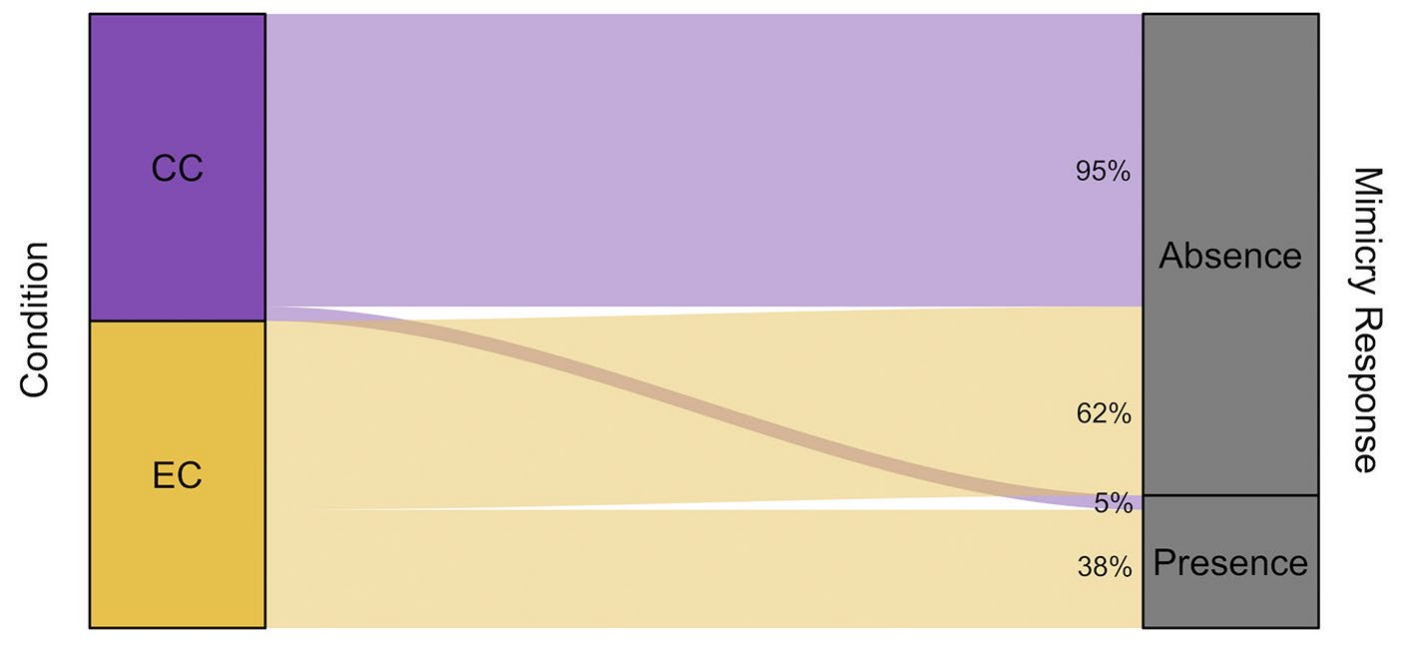
Alluvial plot showing the percentage of mimicry response in the Experimental Condition (EC) and in the Control Condition (CC).

**Fig. 3:**
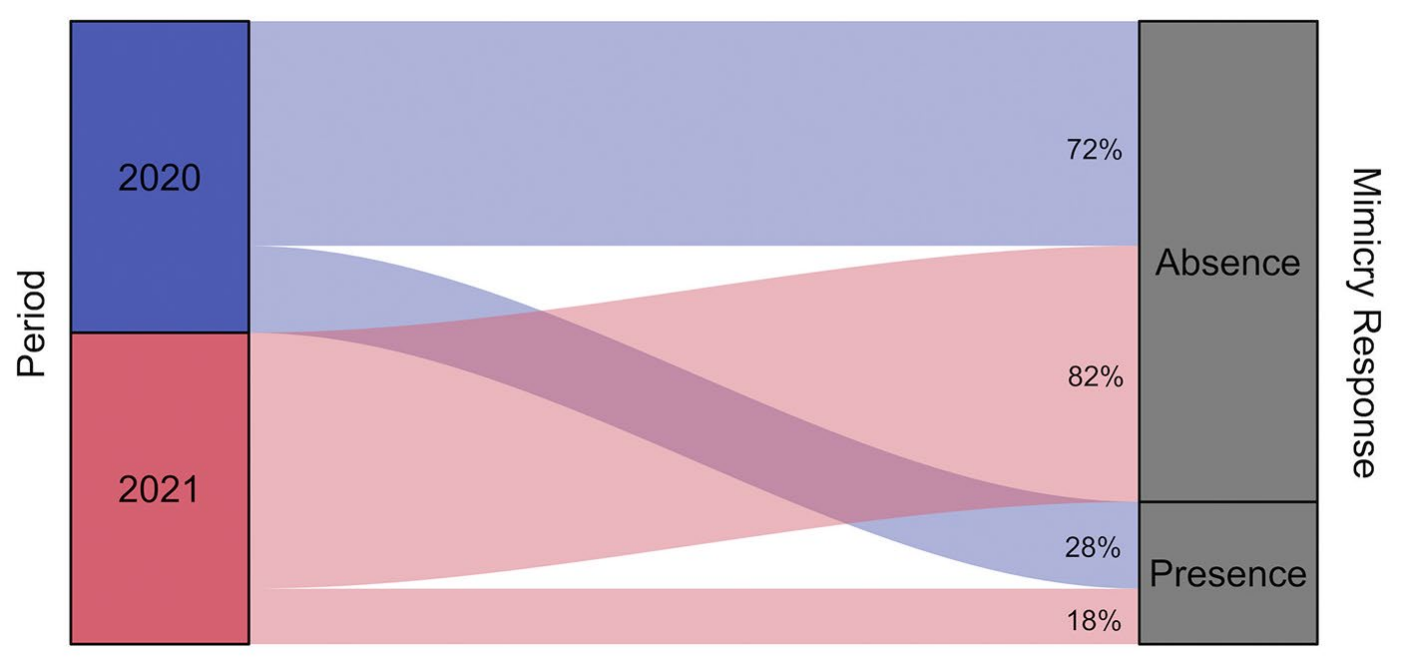
Alluvial plot showing the percentage of mimicry response in the two periods of data collection (2020 and 2021)

**Fig. 4:**
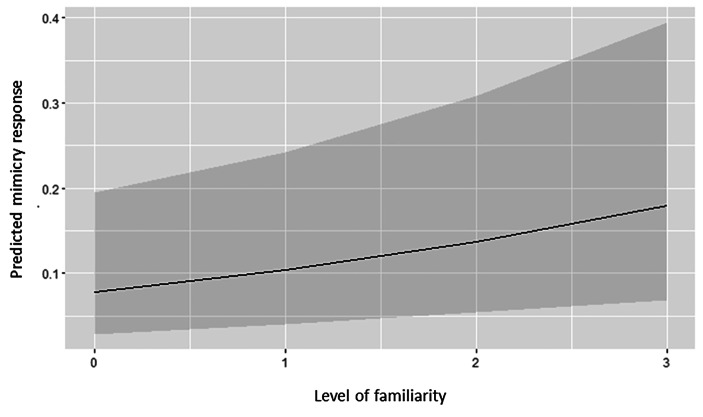
Predicted mimicry response distribution according to the four levels of familiarity clusters (1 = Strangers, 2 = Acquaintances, 3 = Friends, 4 = Kin/Partners).

The model built to investigate the presence of a mimicry phenomenon in the use of smartphones was significantly different from the null model comprising only the random factors (LRT: χ^2^ = 345.13, df = 12, *p* < 0.001). In detail, the fixed factor “condition” had a significant effect on the probability of the mimicry response (Fig. 2). During the EC, on a total of 839 events, the observers mimicked the triggers’ actions 323 times (mimicry frequency = 0.385). During the CC, on a total of 769 events, the observers mimicked the triggers’ actions 36 times (mimicry frequency = 0.047). The fixed effect “pandemic” also had a significant effect, showing that the frequency of mimicry response was higher during the first data collection, immediately after the total lockdown (frequency EC_2020_ = 0.449/CC_2020_ = 0.042; EC_2021_ = 0.300/CC_2021_ = 0.051; Fig. 3). The fixed factor “context” had a significant effect, with the frequency of the mimicry response decreasing in the presence of food. Lastly, the fixed effect “familiarity” also had a significant effect, with an increasing gradient from strangers to kin/partners (Fig. 4).

The details about the fixed factors are shown in Table 1.

**Table 1:** Estimated parameters (Coeff), Standard Error (SE), and results of the likelihood ratio test (χ^2^) of the Generalized Linear Mixed Model (binomial error distribution) investigating the effect of the condition (EC/CC), age class of the trigger and the observer, the sex of the trigger and the observer, the level of familiarity between the trigger and the observer (strangers; acquaintances; friends; kin), the context (presence of food/absence of food), the period of the day (morning, afternoon and night), and the pandemic period on the presence/absence of mimicry. *N*_cases_ = 1,608; *N*_observers_ = 248, N_triggers_ = 60. Variance for the random factors: ID_trigger_ = 0.386, SD = 0.621, ID_observers_ = 0.893, SD = 0.945. Marginal R^2^ = 0.382; marginal R^2^_full model_−marginal R^2^_null model_ = 0.345; conditional R^2^ = 0.555; conditional R^2^_full model_−conditional R^2^_null model_ = 0.500

Fixed Effects	Coeff	SE	χ^2^	Df	*P*
**Intercept**	−2.479	0.539	n/a	n/a	n/a
**Condition** ^a^	2.864	0.214	178.913	1	**< 0.001**
**Day time** ^a^			5.358	2	0.069
Day time [afternoon]	−0.586	0.260			
Day time [night]	−0.533	0.269			
**Context** ^a^	−1.018	0.185	30.271	1	**< 0.001**
**SEX**_**trigger**_^a^	0.732	0.414	3.127	1	0.077
**SEX**_**observer**_^a^	−0.009	0.237	0.001	1	0.971
**AGE**_**trigger**_^a^			3.712	2	0.156
AGE_trigger_ [26-40yrs]	−0.137	0.384			
AGE_trigger_ [41-60yrs]	1.138	0.731			
**AGE**_**observer**_^a^			3.099	2	0.212
AGE_observer_ [26-40yrs]	−0.436	0.256			
AGE_observer_ [41-60yrs]	−0.425	0.354			
**Familiarity** ^a^			7.844	1	**0.005**
**Period** ^a^	−0.388	0.175	4.909	1	**0.027**

^*a*^ Estimate ± SE refers to the difference of the response between the reported level of this categorical predictor and the reference category of the same predictor

### Use of the Phone by the Observers

In 2020, during the EC, the observers who mimicked the trigger’s action took their phone and checked social media (Facebook, Instagram, Whatsapp) in 183 cases out of 187; in 4 cases it was used to check the time (*n* = 3) or to make a call (*n* = 1) (χ^2^ = 350.42; df = 2; *p* = 0.000). During the CC, the observers picked up their phone and checked social media in 3 of 14 cases; in 11 cases they checked the time (*n* = 7), used the camera to take a photo (*n* = 3), or made a call (*n* = 1) (χ^2^ = 4.57; df = 1, *p* = 0.033).

In 2021, during the EC, the observers picked up their phone and checked social media (Facebook, Instagram, Whatsapp) in 135 of 136 cases; in one circumstance the smartphone was used to make a call (χ^2^ = 171.34; df = 1; *p* = 0.000). During the CC, the observers checked social media in 15 of 22 cases; in 7 cases they checked the time (*n* = 4) or took a photo (*n* = 3) (χ^2^ = 2.91; df = 1, *p* = 0.088).

## Discussion

Despite the lack of time depth in this study, the extensive number of behavioral data points analyzed here (1,608 events; 248 people) allowed not only confirmation of the mimicry phenomenon in the use of smartphones independently of the sex and age of the interacting people but also clarified the influence of familiarity and tested additional hypotheses on the effect of the COVID-19 pandemic on the mimicry itself.

Contrary to our expectations, the mimicry response in the use of smartphones was higher immediately after the lockdown (May–September 2020) than a year later (May–October 2021). Since smartphones are one of the few tools that allowed people to keep in touch with others, probably their use during the lockdown translated into a greater sensitivity to be affected by others’ smartphone manipulation. If we depend for long periods on digital communication to maintain contact with others, this could directly affect our perception of social connectedness and modify how we stay in touch with others.

Using a psychological approach, Parasuraman et al. (2017) found that adult subjects mainly use smartphones to contact other people. Our ethological approach also confirms this finding when the use of smartphones resulted from a mimicry phenomenon. Considering both periods of data collection, in 98.45% of cases people affected by the trigger’s action during the Experimental Condition used their smartphones to establish a virtual connection with other people. In this view, using smartphones is not an activity that takes us away from social interactions, it is a different way to interact with others socially, potentially reaching more people simultaneously through “virtual grooming.” In two recent studies, via interviews and questionnaires, researchers found that Italian children and adolescents made more conspicuous use of smartphones during the pandemic than during the pre-pandemic period (Marengo et al., 2022; Serra et al., 2021). Social media (TikTok first, followed by Facebook and Telegram) were the most addictive applications used during the COVID-19 pandemic by school-age adolescents that led to an increase in smartphone addiction (Marengo et al., 2022; Serra et al., 2021). Accordingly, such a transient higher motivation in using smartphones may also have increased the susceptibility to mimicry in adult human subjects, at least in the period immediately after the lockdown. Although we have no comparable data on mimicry in the use of smartphones during the pre-pandemic period, questionnaire data on the spontaneous use of smartphones in American adults indicate that the use of these devices increased during the early stage of the pandemic relative to the previous one (Nguyen et al., 2021). The lower mimicry response that we recorded a year after the forced social isolation can be explained by a gradual restoration of the baseline levels in the use of smartphones.

The expansion of our previous data collection (Maglieri et al., 2021) unveiled the impact of familiarity in fostering the mimicry response to the use of smartphones. This is the first empirical demonstration that social modulation is linked to the expression of the mimicry response in the use of a tool. In particular, we found that people occupying the extreme categories of the familiarity gradient (strangers versus partners/kin) differed in the mimicry response (Fig. 4). This result has also been obtained for other types of mimicry, such as facial expressions, body postures, and mannerisms (Chartrand & Lakin, 2013; Hess et al., 2022; Kavanagh & Winkielman, 2016; Palagi et al., 2020; van Baaren et al., 2006; Baaren et al., 2009). Thus, it seems that the proximate factors (e.g., empathy propensity, de Waal and Preston, 2017) at the basis of the mimicry phenomena in the use of tools (e.g., smartphones) and nonverbal cues can be shared. Since the experimental and control conditions only differed in terms of the gaze of the trigger (Fig. 1), gaze following could be the key factor explaining the linkage between familiarity and mimicry response. As for other body and facial signals, humans are highly sensitive to the eye-gaze direction of conspecifics, possibly because following others’ gaze can provide a rapid inference and reliable information of others’ attention over space (Capozzi et al., 2018; Emery, 2000). Therefore, seeing the trigger’s eyes directed to the smartphones may induce the observer to do the same, although the target of the gaze is a different device. The ability to synchronize visual attention on objects has a wide phylogenetic base, which is present not only in humans (Astor et al., 2021; Driver et al., 1999; Wolf et al., 2016) but also in other primate (Tomasello et al., 2007) and nonprimate species (dogs, Téglás et al., 2012; corvids, Schmidt et al., 2011). Gaze following appears early in life (Dalmaso et al., 2020; Gredebäck et al., 2010), and the resulting synchronization of actions seems to be one of the scaffolds in the formation of social bonding at a large scale (Chartrand & Lakin, 2013; Wolf et al., 2016). Mimicry in manipulating smartphones can be considered an example of joint action that helps reach behavioral synchrony between individuals and that, in the long-term, can be highly effective in activating and modulating social bonding dynamics. This view could explain our finding of the covariation between the mimicry in the use of smartphones and the level of familiarity shared between the mimickee and mimicker.

In conclusion, during the COVID-19 pandemic we carried out a naturalistic experiment on the effect of social isolation on the mimicry response in the use of smartphones. Our results not only confirmed the presence of the mimicry phenomenon but also showed that limited “live” social interactions can modify, at least in the short term, the ways we interact with others by making us more prone to engage in “virtual” social interactions. The bright side of the coin unveiled by our findings is that such an effect seems to dissolve over time.
